# Electrical Conductivity of Glass Fiber-Reinforced Plastic with Nanomodified Matrix for Damage Diagnostic

**DOI:** 10.3390/ma14164485

**Published:** 2021-08-10

**Authors:** Stanislav Stankevich, Olga Bulderberga, Sergejs Tarasovs, Daiva Zeleniakiene, Maria Omastova, Andrey Aniskevich

**Affiliations:** 1 Institute for Mechanics of Materials, University of Latvia, LV-1004 Riga, Latvia; olga.bulderberga@lu.lv (O.B.); tarasov@pmi.lv (S.T.); andrey.aniskevich@pmi.lv (A.A.); 2 Department of Mechanical Engineering, Kaunas University of Technology, 51424 Kaunas, Lithuania; daiva.zeleniakiene@ktu.lt; 3 Polymer Institute, Slovak Academy of Sciences, 845 41 Bratislava, Slovakia; maria.omastova@savba.sk

**Keywords:** glass fiber reinforced plastic, carbon nanotubes, electrical conductivity, micromechanics, damage diagnostic, voltage distribution, interlaminar fracture

## Abstract

The electrical conductivity of glass fiber-reinforced plastic (GFRP) with epoxy matrix modified by multiwall carbon nanotubes (MWCNT) was studied. The electrical conductivity of nanomodified lamina and multi-layered GFRP was investigated on several levels using a structural approach. Components of the electrical conductivity tensor for unidirectional-reinforced monolayer were calculated similarly as in micromechanics using the conductivity of the nanomodified matrix. The electrical conductivity of multilayer composite was calculated using laminate theory and compared with values measured experimentally for various fiber orientation angles. Calculated and experimental data were in good agreement. The voltage distribution measured throughout the laminate allowed detecting the damage in its volume. The electrode network located on the laminate surface could determine the location, quantification, and geometry of the damage in the GFRP lamina modified with MWCNT. Experimental and calculated electrical resistance data for GFRP double-cantilever beam specimens were investigated in Mode I interlaminar fracture toughness test. Results demonstrate that electrical resistance could be successfully used for the diagnostic of the crack propagation during interlaminar fracture of the MWCNT-modified GFRP.

## 1. Introduction

Glass fiber-reinforced plastic (GFRP) is originally an electrically non-conductive structural composite. Electrically conductive fillers, such as multiwall carbon nanotubes (MWCNT), graphene nanoplatelets, 2D carbides MXene, etc., can be incorporated at concentrations of less than 0.3 wt.% into polymer matrixes of fiber-reinforced plastics to produce structural composites with enhanced mechanical performance and electrical conductivity [1,2,3,4,5]. For successful application of such advanced composite with specific electrical conductivity, it needs to be evaluated prior manufacturing process. The infill amount of MWCNT in the modified matrix, reinforcement angle, and stacking sequence of the laminated plate are the most crucial factors influencing the electrical conductivity of the composite [5,6,7]. Structural mechanic approach will be used for electrical conductivity prediction of MWCNT-modified GFRP plate. According to this approach, the addition of conductive MWCNT provided the conductivity of the epoxy matrix. Conductive matrix, in turn, provides conductivity of unidirectional monolayer with non-conductive fibers. Stack of conductive layers oriented in different angles provides conductivity of a multi-layered GFRP laminate. To predict the electrical conductivity of a nanomodified composite, the structural approach was approbated in [6] and additionally developed with various layups in [7]. However, the experimental and calculated electrical conductivity data did not agree for all layouts of composite plates.

Due to gained electrical conductivity, composites possess the ability for strain and damage monitoring [8,9,10,11,12]. As a result, many works were directed to create an internal health-monitoring system for composite structures by in situ measurements of electrical conductivity [8,13,14,15,16,17]. One of the monitoring methods can be performed by measuring the voltage distribution throughout the laminate with following damage detection in its volume. For nanomodified composite, voltage distribution monitoring can be achieved by applying the electrode network directly on top or inside the composite [6,8,18]. However, in most works, only the presence of damage is considered, without paying much attention to its location or geometry.

Fracture toughness plays an essential role in choosing composite materials for constructive applications. Adding carbon nanotubes to a multilayer composite not only helps to achieve the ability to monitor material damage but also significantly enhances its fracture toughness [19,20,21,22]. Damage diagnostic for interlaminar defects was approbated earlier for fiber-based composites [20,23,24,25,26]. The mentioned papers mostly focused on establishing the relationship between resistance and interlaminar crack propagation for double-cantilever beam (DCB) specimens during Mode I (opening mode) interlaminar fracture toughness test. In articles related to this method of detecting damage, experiments were mainly carried out on materials with high electrical conductivity, such as carbon fiber-reinforced plastic. As well, most of the methods were focused on damage monitoring at relatively high deformations. 

Electrical resistance monitoring during structural damage of material gives an opportunity for health diagnostic of complex composite structures during its service time. However, considering the very high interest in the implementation of damage sensing technology inside the composite structures, the reliability of such a technique still remains inconsistent. Many damage diagnostic methods of nanomodified composites have not been thoroughly examined yet for successful approbation in extensively used modern applications.

The main aim of this study was to evaluate the possibility of electrical conductivity monitoring for damage diagnostic of GFRP with MWCNT nanomodified matrix. To achieve this aim following tasks were formulated: (1) To check the possibility of the electrical conductivity prediction of GFRP with nanomodified conductive matrix by the structural approach widely used for prediction of elastic properties of the composites; (2) to evaluate the electrical conductivity response of the nanomodified GFRP plate to a generated damage in its volume via characterization of the voltage distribution throughout the composite plate; (3) to check the correlation between electrical resistance response of nanomodified double-cantilever beam specimens with crack propagation during interlaminar fracture tests of the composite.

This research considered obtained results [6,7], shortcomings, and progress of previous works, and successfully applied the structural approach for prediction of the electrical conductivity of a multilayer composite including its different layup configurations. The location and geometry of the damage diagnostic in the composite plate was brought to the fore as part of the testing. Closer look was taken at the possibility of local diagnosis of damage at small deformations due to the introduction of thin conductive elements into the structure of the composite.

## 2. Materials and Methods

The GFRP composite under investigation was based on unidirectional (UD) glass fabric (GF) supplied by Havel Composites CZ Company Ltd. (Svésedlice, Czech Republic), and two-component epoxy resin system Biresin ^®^ CR122 was supplied by SIKA (Baar, Switzerland). MWCNT NC7000™ supplied by Nanocyl SA (Sambreville, Belgium) was utilized as a stable nanofiller [27,28,29] for epoxy modification and spray-coating. The basic properties of the used materials are presented in Table 1.

### 2.1. Epoxy System Nanomodification

High shear mixer DISPERMAT^®^ LC30 was used to disperse directly added MWCNT in the volume of epoxy resin. A disk blade of 40 mm width at the speed of 10,000 rpm was used to initiate the de-agglomeration process. The mixing procedure was carried out in the cold-water bath to avoid concentrate overheating. For the same reason, four mixing cycles of 10–15 min each were accomplished with 5–7 min pauses in-between. As far as cycles were over, the solution was degassed at a pressure of −0.98 bar and low speed mixed with hardener at 400–500 rpm to avoid excessive air injection in the matrix. The final 10 min degassing cycle was applied prior to specimen manufacturing.

**Figure 1:**
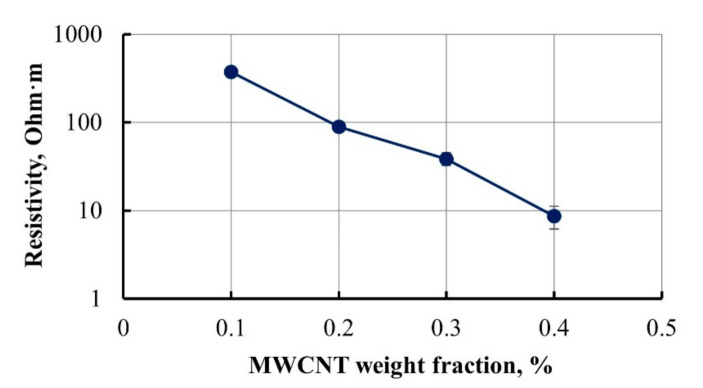
The resistivity of the epoxy matrix modified with different content of MWCNT.

Masterbatches with various MWCNT amounts were prepared to achieve matrix viscosity sufficient for its penetration through layers of GF. The resistivity of the examined nanomodified masterbatches is given in Figure 1.

The resistivity of the epoxy system strongly depends on MWCNT concentration level see Figure 1. At low MWCNTs concentration of 0.1 wt.%, the electrical resistivity of epoxy had a significant drop down to the average of 370 Ω·m. Such electrical resistivity is in the lowest range for MWCNT-modified epoxy compared to other studies [1,3,18]. Resistivity difference up to several orders of magnitude was achieved with increasing of MWCNT weight fraction up to 0.4 wt.%. Further increase of nanofiller concentration was not necessary, as well would be slightly handicapped due to the viscosity rise. The nanofiller concentration of 0.3 wt.% was chosen for composite matrix manufacturing.

**Figure 2:**
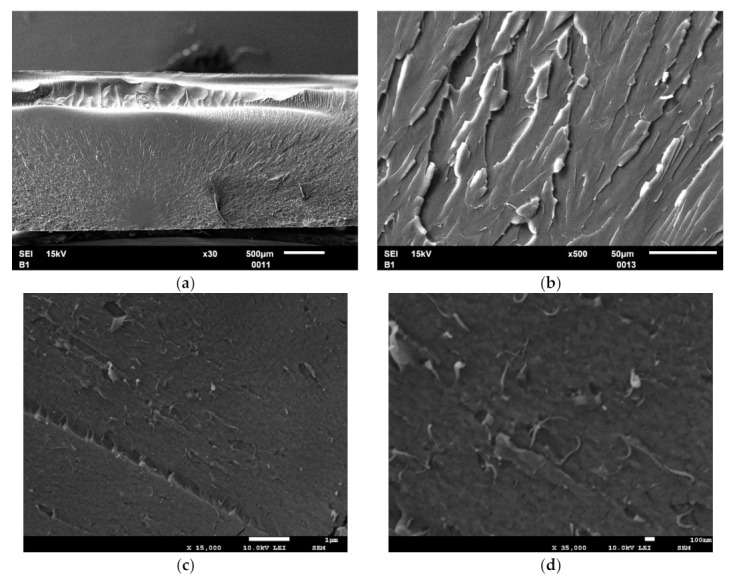
SEM micrographs of fracture surface of 0.2 wt.% MWCNT-modified epoxy at magnifications 30× (**a**); 500× (**b**); 15,000× (**c**); 35,000× (**d**).

The fracture surface of 0.2 wt.% MWCNT-modified epoxy is demonstrated in Figure 2. Separately located nanotubes and small agglomerates can be distinguished in the pictures, meaning that the nanofiller is quite well mixed in the binder. Good nanofiller dispersion contributes to the uniform distribution of electrically conductive tracks in the sample volume.

### 2.2. Unidirectional and Cross-Ply Composite Lamination

The unidirectional-reinforced composite laminate was prepared using the following procedure. GF layers were impregnated one by one with nanomodified epoxy resin between two polyethylene (PE) films. The resin was applied directly to the GF and covered with a top PE layer. The resin was evenly distributed within the GF sheet with randomly directed roller movements applied on the top of the PE film. After preparing the prepreg, they were sequentially laid on a waxed glass base. Once all layers were stacked, peel-ply and breathing net were applied on top of the last GF layer. The prepared stack was sealed using a vacuum bag. The vacuum of −0.98 bar was applied at the room temperature of 20 °C. The additional pressure of ca 0.012 MPa was applied on top of the vacuum bag using weights. In such conditions, the vacuum bag was left for 18 h straight. The post-curing procedure was done according to epoxy system manufacturer recommendations: heating at rate 0.2 °C/min until 110 °C; keeping steady at 110 °C for 10 h; cooling down at rate −0.5 °C/min to avoid unexpected structural distortions due to the thermal shock. The volume fraction of GF in the composite specimens was kept at ca 68%.

## 3. Prediction of the Electrical Conductivity by the Structural Approach

The electrical conductivity is a second rank tensor, and its components for unidirectionally reinforced monolayer were calculated similarly as in micromechanics using the conductivity of the nanomodified matrix. MWCNT-modified matrix is usually considered an isotropic material. A variety of models are applied to estimate its electrical conductivity depending on the content of conductive fillers [7,27,33]. To characterize the electrical conductivity of multi-layered composites, the conductivity of matrix, reinforcement angle, and stacking sequence of the laminated plate should be taken into account. For that reason, the structural approach that is widely used for the prediction of mechanical and thermal properties [34,35] was adopted. According to such an approach, conductivity characterization of the multi-layered composite plate could be considered on several structural levels, from nanomodified epoxy matrix to complete stacked composite laminate.

### 3.1. Micro-Scale of Composite

**Figure 3:**
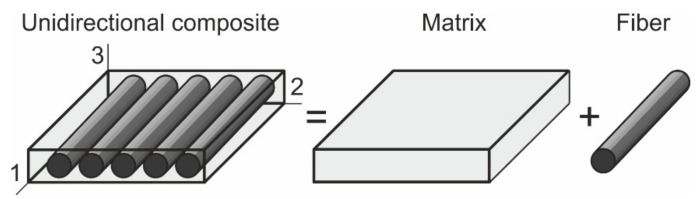
Scheme of a UD composite.

A fiber-reinforced UD composite could be considered a set of long parallel glass fibers embedded in a polymer matrix at the micro-scale level Figure 3 [36].

Considering the transversely isotropic symmetry of UD composite, its tensor of electrical conductivity $\sigma_{ij}$ in main axes of symmetry could be defined as follows:

(1) $$\sigma_{ij}=\left(\begin{array}{ccc} \sigma_{11} & 0 & 0\\ 0 & \sigma_{22} & 0\\ 0 & 0 & \sigma_{33} \end{array}\right)$$

As long as UD GF are lined to axis 1, it was assumed that $\sigma_{22}=\sigma_{33}$ thus two independent components fully define the material. The components $\sigma_{11}$ and $\sigma_{22}$ may be calculated using the rule of the mixture and already known equations from thermal conductivity, diffusivity, etc., [35]:

(2) $$\sigma_{11}=\eta \sigma_{11}^{f}+(1-\eta )\sigma^{m}$$

and

(3) $$\sigma_{22}^{}=\sigma^{m}\left[1+\frac{\eta }{\sigma^{m}/(\sigma_{{}_{22}}^{f}-\sigma^{m})+(1-\eta )/2}\right]$$

where *η* is the volume fraction of glass fibers, $\sigma^{m}$ and $\sigma^{f}=0$ are conductivity for matrix and fibers, respectively.

### 3.2. Monolayer

**Figure 4:**
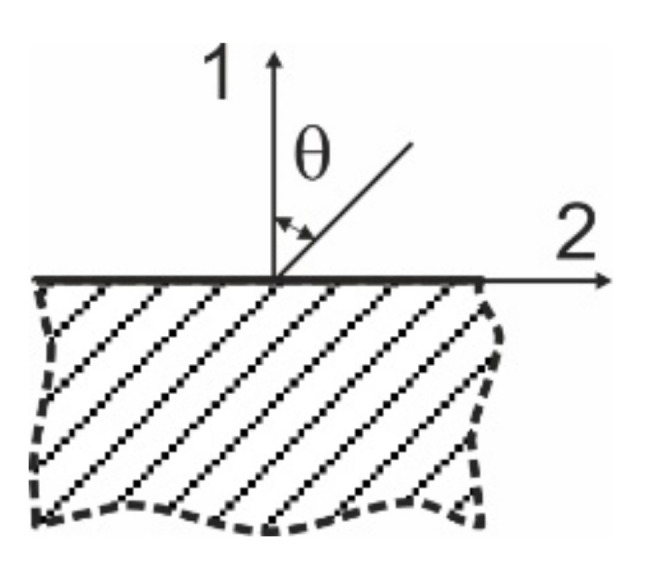
Notation of reinforcement angle in the material axes.

The specific case needs to be evaluated to calculate the conductivity with GFRP orientation at a reinforcement angle *θ* rather than 0°, as shown in Figure 4.

Transformed conductivity tensor looks as following:

(4) $$\sigma {'}_{kl}=\left(\begin{array}{ccc} \sigma {'}_{11} & \sigma {'}_{12} & 0\\ \sigma {'}_{21} & \sigma {'}_{22} & 0\\ 0 & 0 & \sigma {'}_{33} \end{array}\right)$$

where $\sigma {'}_{kl}=\sigma_{ij}\cos(\theta_{ki})\cos(\theta_{lj})$ (short form is used for tensor summation by indexes) with angles $\theta_{2'2}=\theta_{1'1}$. Components of the tensor could be written as

(5) $$\sigma {'}_{11}=\sigma_{11}\cos^{2}\theta +\sigma_{22}\sin^{2}\theta \;$$

(6) $$\sigma {'}_{22}=\sigma_{11}\sin^{2}\theta +\sigma_{22}\cos^{2}\theta \;$$

and

(7) $$\sigma {'}_{12}=\sigma {'}_{21}=(\sigma_{22}-\sigma_{11})\sin\theta \cos\theta$$

Case of *θ* = 45°, the components could be simplified to:

(8) $$\sigma {'}_{11}=\frac{1}{2}(\sigma_{11}+\sigma_{22})\;;\;\sigma {'}_{22}=\frac{1}{2}(\sigma_{22}+\sigma_{11});\;\sigma {'}_{12}=\frac{1}{2}(\sigma_{22}-\sigma_{11})$$

Relative conductivity $\sigma_{rel}$ could be expressed as:

(9) $$\sigma_{rel}=\frac{\sigma {'}_{11}(\theta )-\sigma_{22}}{\sigma_{11}-\sigma_{22}}$$

**Figure 5:**
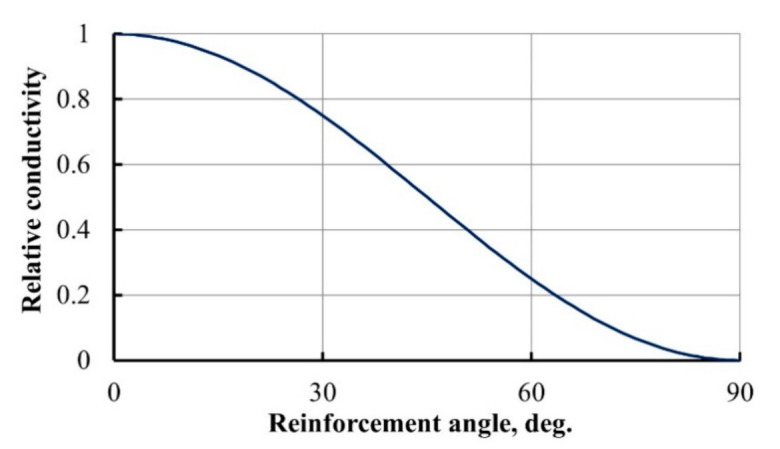
Dependence of relative conductivity on the angle of reinforcement.

The influence of the angle of reinforcement *θ* on composite relative conductivity is given in Figure 5.

As composite reinforcement was rotated from longitudinal to the transverse direction, the relative conductivity was changing gradually from 1 to 0, as could be expected.

### 3.3. Laminate

**Figure 6:**
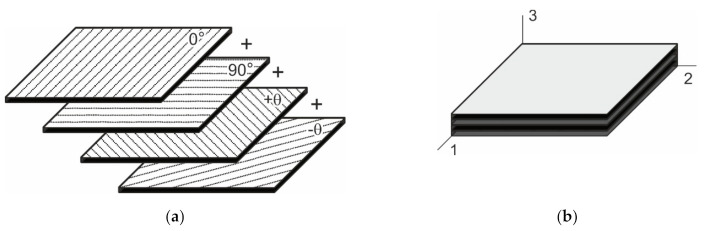
Lamination scheme: (**a**) Stacking of monolayers with angles 0, 90, and ±*θ*°; (**b**) fully stacked laminate.

Stack of conductive layers oriented in different angles provides conductivity of a multi-layered GFRP laminate as presented schematically in Figure 6.

The investigated case represented three layup configurations [0°]_8_, [45°]_8_, and [90°]_8_. For the *N*-layered lamina of thickness *H*, in-plane conductivity ${\bar{\sigma }}_{11}^{'}$ and ${\bar{\sigma }}_{22}^{'}$ was calculated using [7]

(10) $$\bar{\sigma }{'}_{kl}=\frac{1}{H}\sum_{i=1}^{N}h_{i}\sigma {'}_{kl(i)}$$

Taking into account Equations (5)–(7), the tensor components of the lamina are the following:

(11) $$\bar{\sigma }{'}_{11}=\frac{1}{H}\sum_{i=1}^{N}h_{i}(\sigma_{11}\cos^{2}\theta_{i}+\sigma_{22}\sin^{2}\theta_{i})$$

and

(12) $$\bar{\sigma }{'}_{22}=\frac{1}{H}\sum_{i=1}^{N}h_{i}(\sigma_{11}\sin^{2}\theta_{i}+\sigma_{22}\cos^{2}\theta_{i})$$

For the specific layup [*±θ*°]_4_ conductivity tensor components were defined by the following simplified equations [7]:

(13) $$\bar{\sigma }{'}_{11}=\sigma_{11}\cos^{2}\theta +\sigma_{22}\sin^{2}\theta \;\mathrm{and}\;\bar{\sigma }{'}_{22}=\sigma_{11}\sin^{2}\theta +\sigma_{22}\cos^{2}\theta \;$$

If *θ* = ±45°, then:

(14) $$\bar{\sigma }{'}_{11}=\frac{1}{2}(\sigma_{11}+\sigma_{22})\;\mathrm{and}\;\bar{\sigma }{'}_{22}=\frac{1}{2}(\sigma_{22}+\sigma_{11})$$

Thus, Equation (14) allowed calculating conductivity for a specimen cut from a plate with *θ* = ±45° layup.

### 3.4. Verification

To validate the prediction of electrical conductivity by the structural approach, calculated data were compared to the experimental ones on different structural levels. Nanomodified GFRP laminates were cut into 15 smaller specimens with dimensions of 50 × 20 × 3 mm in three separate groups with reinforcing angles 0, 90, and 45°. Electrical conductivity evaluation of those specimens was done using 2- and 4-point probe methods (denoted as 2 PPM and 4 PPM, respectively), which are mentioned in various papers [37,38]. Comparison of these two methods allows being aware of the effect of contact resistance between specimen and electrodes. Electrical contacts were created using conductive silver paint ELECTRON 40 AC. Multimeter Tektronix DMM 4020 was used for measurements.

**Figure 7:**
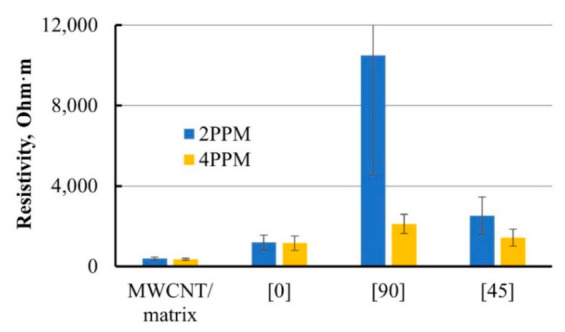
Comparison of 2- and 4-point probe methods (2 PPM and 4 PPM, respectively) for resistivity measurement of 0.3 wt.% MWCNT-modified matrix and its based composite bars with reinforcing angles 0°, 90°, and 45°.

The measurements by 2 PPM and 4 PPM were done for all three specimen groups of composite and nanomodified epoxy matrix. The results are presented in Figure 7. A two-fold difference in data obtained by those two methods was observed for some specimens, see Figure 7. Using 4 PPM, smaller electrical resistivity was obtained for all bar-type composite specimens with various angles of reinforcement. The 4 PPM method was used for resistivity measurement in all experiments further on where absolute values were necessary.

**Figure 8:**
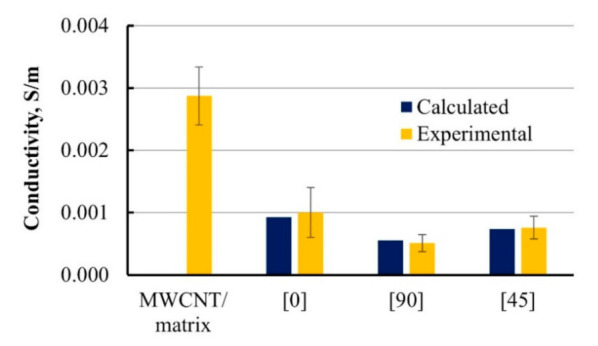
Calculated and experimental conductivity of UD GFRP with 0.3 wt.% MWCNT-modified epoxy matrix and its based composite bars with reinforcing angles 0°, 90°, and 45°.

Electrical conductivity data calculated by Equations (11) and (12) were compared with experimentally acquired ones using the four-probe method in Figure 8.

Noticeable electrical anisotropy was observed for UD GFRP specimens modified by MWCNT due to the orientation of non-conductive fibers. Electrical conductivity values for composite along the fibers were two times higher than those for transverse direction. Even higher degree of anisotropy of one-order of magnitude was achieved in [15]. Such behavior was expected and could be described with a higher amount of obstacles for current to flow around created by transverse-orientated fibers. Calculated and experimental electrical conductivity data for various fiber orientation angles were in good agreement.

## 4. Voltage Distribution in Damaged Nanomodified Composite Plate

**Figure 9:**
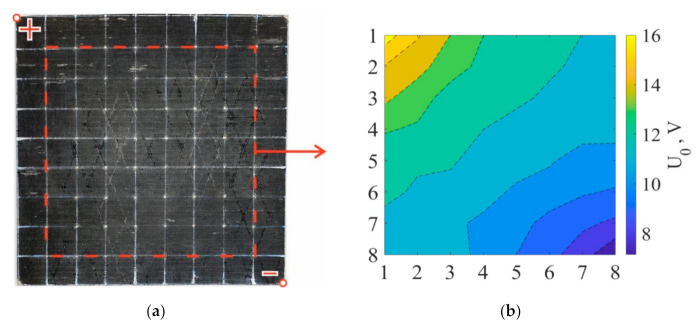
GFRP/MWCNT [0°, 90°]_4_ undamaged composite plate (**a**) and experimentally obtained voltage distribution (**b**). Vertical and horizontal numbers from 1 to 8 define the coordinates of electrodes on the composite plate.

To evaluate the electrical conductivity usage for composite plate’s damage diagnostic, MWCNT-modified composite plate with dimensions of 235 × 235 mm was utilized. The reinforcement orientation of [0°, 90°]_4_ was chosen for an equal conductivity in the *x* and *y* directions. For characterization of voltage distribution throughout the laminate, an electrode network of 8 × 8 contacts was formed by applying conductive paint on the polished top surface of the composite plate. The voltage of 20 V was applied between the diagonally opposite corners of the plate (upper left and lower right corners in Figure 9a).

The voltage distribution was obtained by the voltage drop measuring on each contact for the initial undamaged state (later called case 0 in the text and Figure 9b).

**Figure 10:**
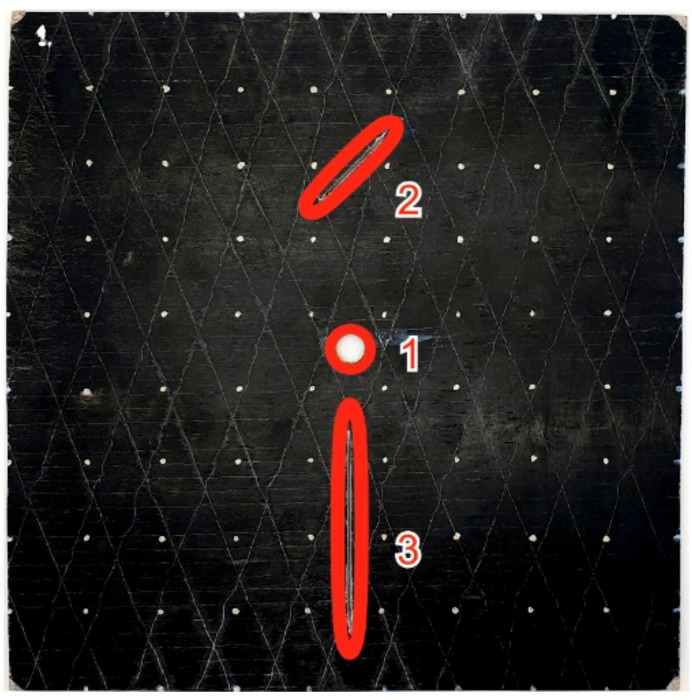
The GFRP/MWCNT composite plate with defects: hole (case 1) and notches (cases 2 and 3). Numbers show the sequence of the created defects.

Three different cases of damage were consecutively examined and presented in Figure 10: (1) A circular hole of diameter 9.8 mm in the middle; (2) a notch of 31 mm length and 1.8 mm width was added; (3) and one more notch of 70 × 1.8 mm was added to the plate.

Using the network of 64 contacts, the distribution of voltage in the GFRP plate was measured experimentally at each sequential damage state. The voltage field on contact points was registered at each state and compared with the previous one to observe the transformation of voltage distribution caused by the generated damage.

**Figure 11:**
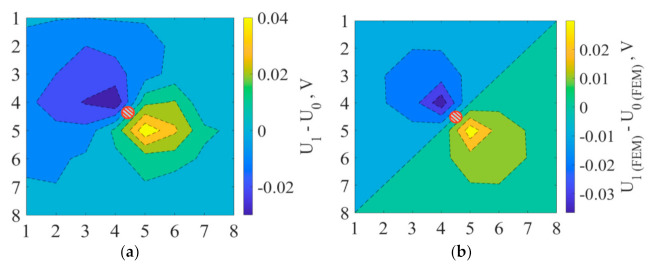
Experimental (**a**) and calculated using FEM (**b**) voltage distribution difference for the plate with the hole (case 1) and its undamaged state (case 0). Damaged areas are red line highlighted. Vertical and horizontal numbers from 1 to 8 define the coordinates of electrodes on the composite plate.

The distribution of the electric field potential in a plate for all damage states was simulated using two-dimensional isotropic static electric analysis by finite element method (FEM) in Ansys. The FEM was used to test and verify the accuracy of the electric potential measurements. Therefore simple 2D model with isotropic electric properties was applied. For evaluation of damage characterization capability by voltage distribution monitoring, experimental data were compared with the simulation model, as shown in Figure 11, for example, damage case (1), where $U_{i}$ with *i* = 0, 1, 2, 3 corresponds to the *i*-th state. To compare the disturbed electrical potential isolines, simulated results were recorded at the 64 points with the same coordinates and applied voltage as in the experiment.

As seen from Figure 11, experimental and calculated voltage distribution affected by created defect had similar geometry and absolute values.

**Figure 12:**
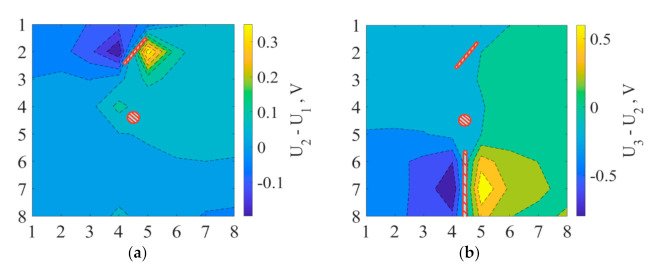
Experimentally obtained voltage distribution difference between the cases (2) and (1) (**a**), cases (3) and (2) (**b**). Damaged areas are red line highlighted. Vertical and horizontal numbers from 1 to 8 define the coordinates of electrodes on the composite plate.

At every damage state of the plate, the highest voltage spikes occurred on the sides of the aperture along the potential lines. As seen from Figure 11 and Figure 12, equipotential lines’ front in the nearest area of the damage mimicked the geometry of the aperture. Thus, voltage distribution maps for all damage cases showed the aperture’s proper position and geometry.

Relative error *δ* between experimental and calculated data for different damage cases were found using

(15) $$\delta =\frac{1}{64}\sum_{i=1}^{64}\frac{\left|U_{\mathrm{FEM}}^{i}-U_{\exp}^{i}\right|}{U_{\exp}^{i}}\cdot 100\%$$

where *i* is a consecutive number of composite plate’s electrode. The average relative error of 6.2 ± 0.2% for all states of the composite plate showed an excellent resemblance between calculated and experimental voltage data. Therefore, the generated damage of a plate could be predicted by the transformation of the voltage distribution in the laminate.

## 5. Delamination Crack Monitoring in Double-Cantilever Beams

Electrical resistance response was used to characterize the interlaminar fracture of the composite. Two types of DCB specimens were used for this: (1) With nanomodified conductive matrix—electrical conductivity through all volume of a specimen was provided in this case; (2) with a neat matrix and MWCNT-modified interleave that provided conductivity of the thin layer only in the middle of a specimen. The crack during the test was intentionally initiated in this layer exactly.

DCB specimens were prepared according to the ASTM D5528 standard with dimensions of 125 × 25 × 3.0 mm. DCB specimens were cut from the 8-layered UD GFRP plate prepared by the laminate stacking technique described in subsection 0. Polytetrafluorethylene (PTFE) film with a thickness of 8 μm was added between the 4th and 5th layers that initiated interlaminar crack inside of the DCB specimen.

### 5.1. Double-Cantilever Beam with Nanomodified Matrix

**Figure 13:**
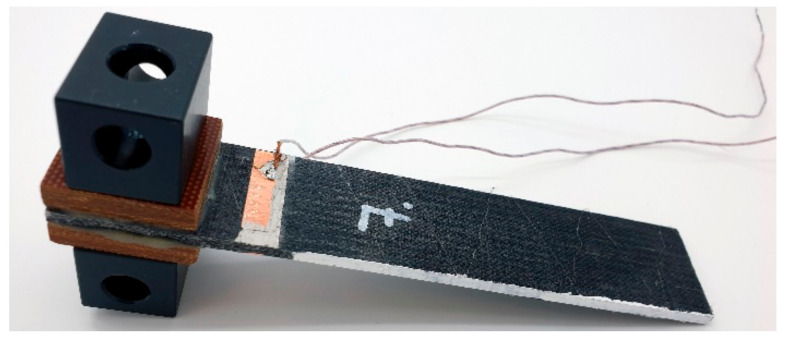
MWCNT-modified GFRP DCB specimen with attached copper electrodes, two glued tabs, and screwed loading blocks.

Tests were performed according to the ASTM D5528 standard on a Zwick universal testing machine with a load cell of 2.5 kN at a constant crosshead speed of 5 mm/min. Considering the conductive matrix of the MWCNT-modified GFRP, copper tape electrodes were placed directly on top of both polished sides of the DCB, Figure 13.

**Figure 14:**
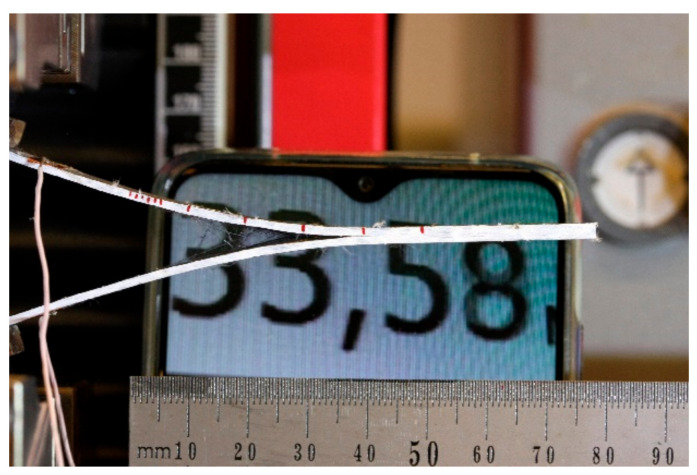
DCB specimen of GFRP with built-in foil electrodes during Mode I interlaminar fracture toughness test.

The electrical resistance of DCB specimens was monitored by the 2-point probe method during Mode I interlaminar fracture toughness test. Contact wires were welded to each side of the specimen to copper tape electrodes. Wires were connected to Zwick machine input. The electrical resistance of each specimen was measured prior to and during the crack opening phase, as shown in Figure 14.

**Figure 15:**
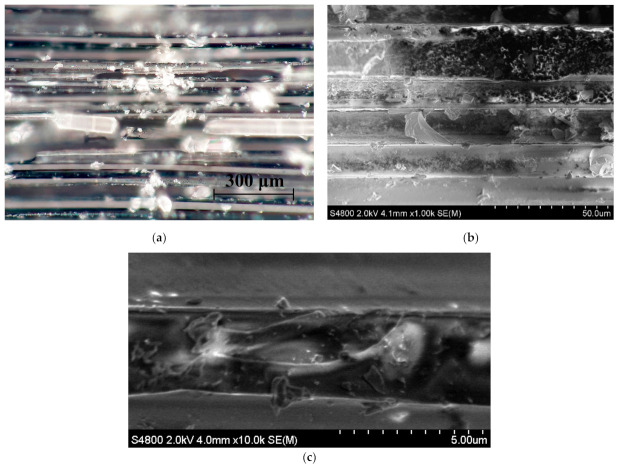
Optical (**a**) and SEM (**b** and **c**) micrographs of fracture surface of nanomodified epoxy DCB sample after test. Magnifications: 500× (**a**); 1000× (**b**); 10,000× (**c**).

Optical and SEM images of DCB sample’s fracture surface was obtained after testing, see Figure 15. Pieces of fractured epoxy can be still seen sticking to GF even after the test.

Fiber-matrix interface failure is the dominant failure mode in DCB specimens [24]. From Figure 15b it can be noticed that epoxy resin was locally stripped from the GF surface. However, nanotubes can be still seen holding to GF surface with some amount of matrix combined, see Figure 15c. MWCNT provides conductive tracks in the specimen volume but also helps the matrix to adhere to the fiber surface even under load. Fracture toughness is known to increase by 5–15% for composites with 0.1–0.5 wt.% of MWCNT [19,21].

**Figure 16:**
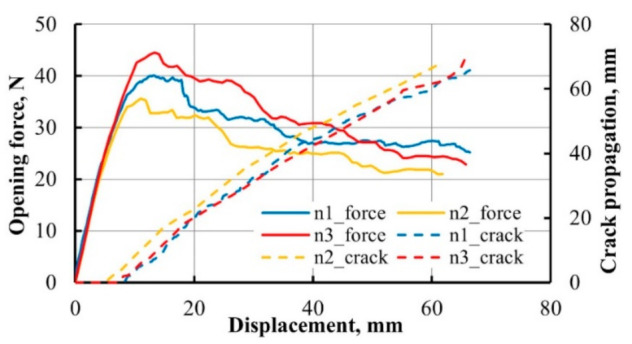
Opening force and crack propagation of DCB specimen vs. machine’s grips displacement.

Force-displacement data of three DCB specimens with nanomodified matrix are presented in Figure 16. Crack propagation distance was acquired from the photos obtained each 3 s during the test and synchronized with Zwick machine data in time.

**Figure 17:**
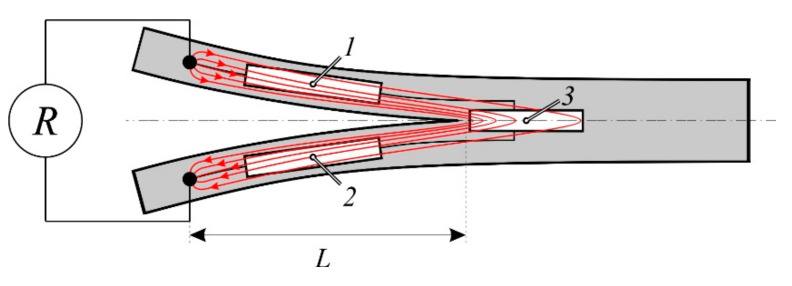
Schematic representation of DCB specimen during the Mode I interlaminar fracture toughness test. *L* is the distance from connected electrodes to the tip of the crack. Sections *1*, *2*, and *3* represent the resistive volume of the specimen as sequentially connected resistors, and red lines schematically show the current flow.

A simplified model of three sequentially connected resistors was proposed to predict the crack propagation using electrical resistance of DCB specimen, see Figure 17.

Full resistance *R* measured from electrode to electrode during a test was expressed as a sum of all resistors

(16) $$R(t)=R_{1}(t)+R_{2}(t)+R_{3}(t);\;R_{1}(t)=R_{2}(t)$$

where *R*_1,2_ is the resistance of delaminated sections *1* and *2*, which considered to be equal due to their identical geometry:

(17) $$L(t)=L_{1}(t)=L_{2}(t);\;S_{1}=S_{2}=const$$

where *L*_1,2_ and *S*_1,2_ are the length and cross-section of sections *1* and *2*, respectively. The resistance of sections *1* and *2* could be calculated using resistivity *ρ*_11_ of the composite along the fiber direction:

(18) $$R_{1,2}=\frac{\rho_{11}L(t)}{S}$$

Resistivity *ρ*_11_ was measured independently for each specimen when sections *1* and *2* were fully separated after the test. The delaminated faces of the specimen open away from each other during the test, elongating the crack and sections *1* and *2*. Within the presented model, section *3* was considered to have a constant resistance because it is supposed that the current with the highest density is located in the relatively small zone closest to the crack tip, as illustrated in Figure 17 with red lines. This zone in front of the crack propagates together with the crack, but the current density and resistivity remain unchanged in this area almost until the complete delamination of the specimen. Therefore, the resistance of section *3* could be found from the initial state of the specimen before the test as

(19) $$R_{3}=R(0)-2\frac{\rho_{11}L(0)}{S}$$

Keeping in mind that *R*_3_ was assumed to be constant, total resistance can be fully defined with geometric parameters of sections *1* and *2*. In this manner, total resistance during the delamination test could be calculated as follows:

(20) $$R(t)=2\frac{\rho_{11}L(t)}{S}+R_{3}$$

**Figure 18:**
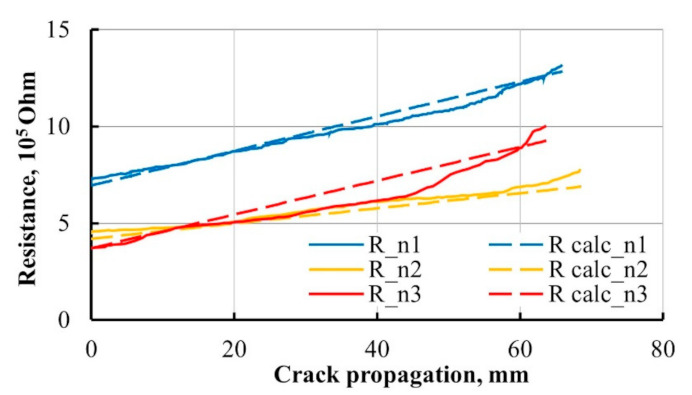
Experimental (solid line) and calculated (dashed line) electrical resistance of MWCNT-modified 8-layered GFRP composite DCB during Mode I interlaminar fracture toughness test.

For verification of the presented model, experimental and calculated resistance data were compared with reference to DCB specimen crack propagation and given in Figure 18.

Calculated electrical resistance showed a similar increase as experimental during the crack propagation. The presented simplified model appeared to have adequate prediction repeatability through various test specimens. The difference between resistance absolute values for different specimens was caused by their resistivity variation before the tests.

### 5.2. DCB with Nanomodified Interleave

**Figure 19:**
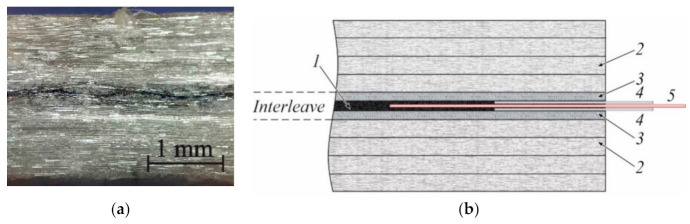
Eight-layered UD GFRP composite plate with the interleave. Micro photo of the longitudinal cross-section with magnification 200× (**a**) and the laminate structure (**b**). The spray-coated layer of MWCNT (*1*); UD glass fibers (*2*); woven glass fibers (*3*); aluminum foil electrode (*4*); PTFE film (*5*).

With volumetric matrix nanomodification, only integral damage diagnostic can be provided in all specimen’s volume. Thus, the whole structural element is a single sensor, and in that case, damage localization is impossible. The situation changes with the embedding of multiple conductive sensors to the structural element. This can be realized via the implementation of several conductive interleaves. Interleave can be created by spray-coating of conductive nanoparticles during manufacturing of the structural element. To verify this concept and develop technology of interleaved composite manufacturing, GFRP plate with a neat epoxy matrix and single conductive interleave was produced. Interleave consisted of two woven GF layers with an area density of 130 g/m^2^ supplied by Havel Composites CZ Company Ltd., and a spray-coated layer of MWCNT between them. The spray-coating procedure was carried out using MWCNT-acetone solution by spray-gun SAP-CR 0.2 supplied by STAR^®^, with a nozzle size of 0.2 mm at the pressure of 0.1 MPa. The solution was prepared using an ultrasonic probe sonicator at 120 W for 1 h with three 5-min pauses in-between. The amount of MWCNT in mass per area was held at 2.3·10^−4^ mg/mm^2^ during the spray-coating procedure. Each of the two GF layers was one side spray-coated, and a foil electrode was attached at one end. These two layers were stacked with sprayed sides face to face, as presented in Figure 19. PTFE film was placed in between for the crack initiation and proper initial resistance monitoring.

**Figure 20:**
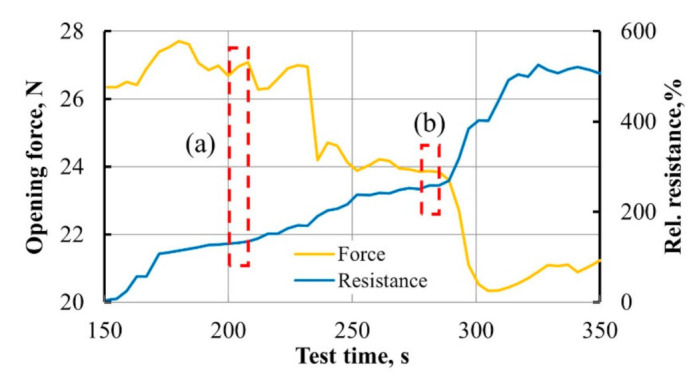
The opening force *F* and relative resistance *R_rel_* in time during crack propagation for neat epoxy-based DCB specimens with MWCNT spray-coated interleave. Red sectors (**a**) and (**b**) are examined in Figure 21.

Electrical resistance data of DCB between two electrodes were collected by the Zwick machine during Mode I interlaminar fracture toughness test and synchronized in time with opening force applied to the specimen. Combined force and relative resistance data in time interval 150–350 s during the crack propagation are given in Figure 20.

Correlation coefficient *r* [39] was calculated to evaluate the relationship between the force applied to DCB specimen and its resistance.

(21) $$r=\frac{\Sigma (F(t)-\bar{F})(R_{rel}(t)-{\bar{R}}_{rel})}{\sqrt{\Sigma {\left(F(t)-\bar{F}\right)}^{2}\Sigma {\left(R_{rel}(t)-{\bar{R}}_{rel}\right)}^{2}}}$$

(22) $$R_{rel}(t)=\frac{R(t)-R(t=0)}{R(t=0)}\cdot 100$$

where *F*(*t*) is the opening force measured in time, *R_rel_*(*t*) is relative resistance, $\bar{F}$ and ${\bar{R}}_{rel}$ are mean values for opening force and relative resistance within the examined time interval.

The correlation value of *r* = −0.945 indicates a very strong negative connection between the two processes. The applied force caused DCB delamination process, which showed a great effect on specimen resistance. Some unsynchronized variations between the two parameters were observed during the experiment. This potentially may have occurred due to delamination of the conductive sprayed layer and the specimen. Therefore conductive paths were not broken even during the crack propagation.

**Figure 21:**
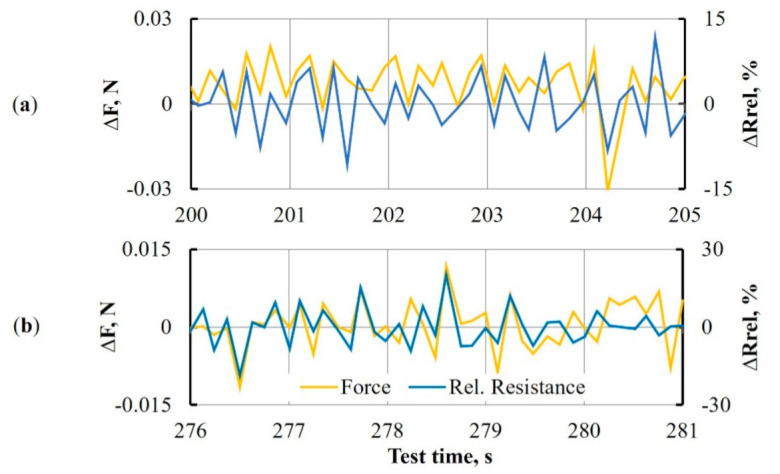
Parameters ∆*F* and ∆*R_rel_*_._ in time intervals of 200–205 (**a**) and 276–281 s (**b**) during crack propagation for neat epoxy-based DCB specimen with MWCNT spray-coated interleave.

The more detailed characterization was provided at much shorter time intervals to estimate the resistance response to small force adjustments. For that reason, two random intervals of 5 s were taken for examination. Two parameters $\Delta F=F_{n}-F_{n-1}$ and $\Delta R_{rel.}=R_{rel.\;n}-R_{rel.\;n-1}$ were analyzed and presented in Figure 21.

Obtained results showed a good connection between force and resistance values during the experiment at small force adjustments. Each force jump that occurred in the crack propagation, even within the margins of ±0.015 N, was followed by a jump of resistance. As could be seen from Figure 21, resistance response became higher during the test.

**Figure 22:**
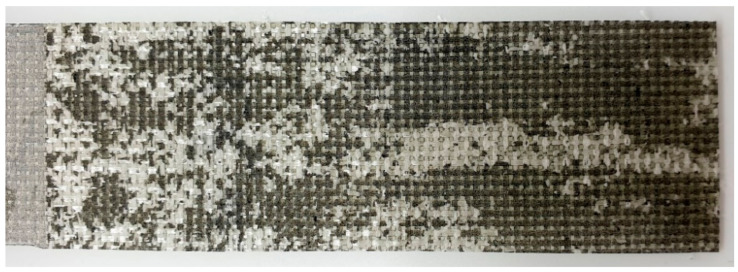
The surface of fractured epoxy-based DCB specimen with MWCNT spray-coated interleave after delamination.

Such behavior could be described by a large number of defects in the conductive layer after delamination, see Figure 22, which led to resistance rise. Therefore, following crack propagation would have a more substantial effect on resistance until the complete conductivity loss. Figure 22 illustrates the degradation of the MWCNT conductive layer during the test. Examining the fracture surface, it can be seen that the surface of the electrically conductive layer is not uniform after the degradation process. Intermittent destruction of the conducting layer leads to sharp jumps of resistance, and at the same time, a huge decrease of electrical conductivity occurred up to its complete loss. To avoid such sudden signal interruption, the thickness and strength of the nanofiller layer should be improved to conduct the signal until the total delamination. For this, upper and lower half of the interleave could be both sides spray-coated or fully dipped in a MWCNT solution. In that case, the inner side of each GF will be fully covered with nanotubes, even during the test.

## 6. Conclusions

1. It was experimentally proved that electrical conductivity monitoring could be successfully used for open and interlaminar damage diagnostic of the GFRP lamina with the MWCNT-doped polymer matrix.
2. The electrical conductivity of the GFRP composite was experimentally and theoretically considered on different structural levels. The addition of conductive MWCNT provided the conductivity of the epoxy matrix. Conductive matrix, in turn, provides conductivity of unidirectional monolayer with non-conductive fibers. Stack of conductive layers oriented in different angles provides conductivity of a multi-layered GFRP laminate. Well-known equations were applied to calculate the conductivity of the composite on these structural levels. Calculated and experimental electrical conductivities of multi-layered GFRP with MWCNT-modified epoxy matrix are in good agreement.
3. Determining the location, quantification, and geometry of the damage in the MWCNT-modified GFRP lamina can be performed by monitoring the voltage distribution throughout the composite plate. The shape of equipotential voltage lines near the defect in the plate was precisely mimicking the geometry of the defect. Furthermore, voltage distribution in damaged GFRP plate calculated by FEM showed an excellent resemblance with the experimental data for all damage states.
4. Monitoring the electrical resistance can be successfully used to control the crack propagation, as shown for interlaminar fracture tests of the GFRP with epoxy matrix modified by MWCNT in volume. A simple model was proposed to calculate the volume resistance of the DCB specimens in the tests. Calculated and experimental electrical volume resistance showed similar behavior during the whole time interval of the crack propagation.
5. Experimental results prove that adequate damage localization in the GFRP plate was implemented by introducing thin conductive MWCNT-based interleave in the mid-plane of the composite. Furthermore, obtained results indicated a very strong negative correlation between the opening force, the interleave resistance, and the crack propagation.

## Figures and Tables

**Table 1:** Basic properties of used materials for composite preparation by manufacturers’ data.

Material	Parameter	Units	Value
Epoxy resin Biresin ^®^ CR122	E-Modulus	GPa	2.8 [30]
MWCNT NC7000™	Volume resistivity	Ω·cm	1·10^−4^ [31]
	Avg. diameter	10^−9^ m	9.5 [31]
	Avg. length	10^−9^ m	1500 [31]
Glass fabric by Havel Composites	Area density	g/m^2^	500 [32]

## Data Availability

Study did not report any data.
